# Matching population diversity of rhizobial *nod*A and legume *NFR5* genes in plant–microbe symbiosis

**DOI:** 10.1002/ece3.5556

**Published:** 2019-08-30

**Authors:** Anna A. Igolkina, Georgii A. Bazykin, Elena P. Chizhevskaya, Nikolai A. Provorov, Evgeny E. Andronov

**Affiliations:** ^1^ ARRIAM, All‐Russia Research Institute for Agricultural Microbiology Pushkin Russia; ^2^ Peter the Great St. Petersburg Polytechnic University Saint‐Petersburg Russia; ^3^ Center for Life Sciences Skolkovo Institute of Science and Technology Moscow Russia; ^4^ Laboratory for Molecular Evolution Kharkevich Institute of Information Transmission Problems of the Russian Academy of Sciences Moscow Russia; ^5^ Saint‐Petersburg State University Saint‐Petersburg Russia; ^6^ Dokuchaev Soil Science Institute Moscow Russia

**Keywords:** evolutionary molding, *NFR5*, *nod*A, phylogenetic congruence, rhizobium–legume symbiosis, topological diversity

## Abstract

We hypothesized that population diversities of partners in nitrogen‐fixing rhizobium–legume symbiosis can be matched for “interplaying” genes. We tested this hypothesis using data on nucleotide polymorphism of symbiotic genes encoding two components of the plant–bacteria signaling system: (a) the rhizobial *nod*A acyltransferase involved in the fatty acid tail decoration of the Nod factor (signaling molecule); (b) the plant *NFR5* receptor required for Nod factor binding. We collected three wild‐growing legume species together with soil samples adjacent to the roots from one large 25‐year fallow: *Vicia sativa*, *Lathyrus pratensis*, and *Trifolium hybridum* nodulated by one of the two *Rhizobium leguminosarum* biovars (*viciae* and *trifolii*). For each plant species, we prepared three pools for DNA extraction and further sequencing: the plant pool (30 plant indiv.), the nodule pool (90 nodules), and the soil pool (30 samples). We observed the following statistically significant conclusions: (a) a monotonic relationship between the diversity in the plant *NFR5* gene pools and the nodule rhizobial *nod*A gene pools; (b) higher topological similarity of the *NFR5* gene tree with the *nod*A gene tree of the nodule pool, than with the *nod*A gene tree of the soil pool. Both nonsynonymous diversity and Tajima's *D* were increased in the nodule pools compared with the soil pools, consistent with relaxation of negative selection and/or admixture of balancing selection. We propose that the observed genetic concordance between *NFR5* gene pools and nodule *nod*A gene pools arises from the selection of particular genotypes of the *nod*A gene by the host plant.

## Graphical abstract

Interactions between coevolving organisms are described by several theoretical models (e.g., Red Queen, Red King, etc.); however, the underlying factor that causes the implementation of different models is still unclear. This problem is of large importance for understanding co‐evolutionary processes. The present manuscript revealed the possible underlying effect in view of matching population diversities on the symbiosis between Rhizobium species and legumes, and we referred this effect as to the “evolutionary moulding effect.”

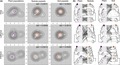

## 1 INTRODUCTION

One of the earliest studied types of symbiosis, the host–parasite interaction, was described by Flor's Gene‐for‐Gene concept (Flor, 1971, 1942) and, in fact, the first mathematical model of coevolution was explicitly based on the assumption of a Gene‐for‐Gene (GFG) interaction (Mode, 1958; Thompson & Burdin, 1992). Further analyses of host–parasite interactions revealed concepts, namely matching‐allele (MA) (Frank, 1994), inverse‐matching‐allele (IMA) (Otto & Michalakis, 1998), and inverse‐gene‐for‐gene (IGFG) (Fenton, Antonovics, & Brockhurst, 2009), which together with the GFG represent the opposite end of the same continuum of host–parasite specificity (Agrawal & Lively, 2002). These theoretical concepts, initially developed for antagonistic systems, found their reflection also in mutualistic symbiotic systems (Cregan, Sadowsky, & Keyser, 1991; Lewis‐Henderson & Djordjevic, 1991; Parker, 1999; Sachs, Essenberg, & Turcotte, 2011; Sadowsky et al., 1991). One of the possible consequences of the above‐mentioned concepts is that matching between symbionts could be observed not only on the gene sequence level but also on the population structure level. We proposed that coordinated population diversity of symbionts can be a significant aspect of symbiotic interactions in a row with the difference in evolutionary rates between interacting species as considered in the Red Queen and the Red King dynamics (Bergstrom & Lachmann, 2003; Pal, Maciá, Oliver, Schachar, & Buckling, 2007; Paterson et al., 2010; Van Valen, 1973).

Previous studies have demonstrated the matching population diversities of symbionts. For example, the analysis of the symbiosis between *Neorhizobium galegae* and its host plant *Galega* indicated correspondence of population diversity levels between microsymbionts and the host *Galega* species (Andronov et al., 2003; Österman et al., 2011). In particular, a more genetically diverse *Galega orientalis* population harbors a more diverse root nodule rhizobial population, while its less diverse sympatric counterpart *Galega officinalis* forms symbiosis with a less diverse rhizobial population. This observation is related to the well‐studied phenomenon of shaping the genetic structure of the rhizobial population through the selection of specific rhizobial genotypes by the host plant (Depret & Laguerre, 2008; Heath & Tiffin, 2007; Laguerre, Louvrier, Allard, & Noelle, 2003; Paffetti et al., 1998). Moreover, it has been shown that the topology of the *nod*A gene tree follows the corresponding host plant tree more strictly than the 16S rRNA‐based rhizobial phylogeny (Dobert, Breil, & Triplett, 1994; Suominen, Roos, Lortet, Paulin, & Lindstro, 2001). Therefore, we expect that the interplay of symbiotic populations leads to concordance between the diversity levels in their symbiotic genes.

**Figure 1:**
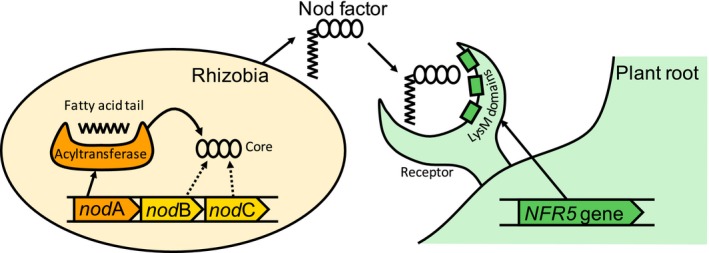
A part of the signal transduction system that governs the rhizobium–legume symbiosis. The rhizobial *nod*A gene encodes the acyltransferase that participates in the attachment of the hydrophobic long‐chain fatty acid tail to the Nod factor core. Plant *NFR5* gene encodes the symbiotic receptor recognizing the rhizobial Nod factor followed by symbiosis formation

In this study, we focused on two symbiotic genes that can be considered interacting as both encode the essential components of the rhizobium–legume signaling system; these are associated with each other through a lipochito‐oligosaccharide called Nod factor (NF) (Figure 1). The first component is the rhizobial *nod*A gene which encodes an acyltransferase enzyme essential in NF biosynthesis, specifically in the attachment of the long‐chain fatty acid tail to the oligosaccharide backbone (Dénarié, Debellé, & Promé, 1996; Esseling & Emons, 2004; Oldroyd, 2013). The second component is one of the plant symbiotic receptor genes, *NFR5*, which is a homologue of *LjNFR5*, *MtNFP*, *PsSym10* genes. Its product recognizes NFs (signaling molecules) by three extracellular LysM domains and triggers the formation of root nodule primordia giving the green light to the process of bacterial infection (Oldroyd, 2013). The NFs are major determinants of host specificity: rhizobia produce NFs with different structures, and host plants percept only those NFs that have a certain composition (Mergaert & Montagu, 1997). The variation of NFs structure is observed not only between rhizobia species but also at the intra‐species level (Spaink, 2000); one rhizobia species produces a mixture of NFs that vary in the fatty tail modifications. As proposed, the *nod*A product can vary in its fatty acid specificity, thus contributing to the bacterial host range (Dénarié et al., 1996; Moulin, Béna, & Stępkowski, 2004; Ritsema, Wijfjes, Lugtenberg, & Spaink, 1996; Roche et al., 1996). It is logical to assume that the *nod*A gene diversity in a rhizobial population can reflect the structural variation of NFs produced by this population. Indeed, it has been shown that minor differences in the structure of fatty acids tail can affect intra‐species host specificity (Li et al., 2011). On the host plant side, NFs are recognized by high‐affinity legume receptors (Broghammer et al., 2012; Moulin et al., 2004). Studies on the model legumes revealed *NFR5* as one of the major receptors to percept NFs (Radutoiu et al., 2007). Mutant analysis showed that single amino acid differences in one domain of the *NFR5* receptor change recognition of NF variants (Broghammer et al., 2012; Radutoiu et al., 2007). Such mediation of NFs between rhizobial *nod*A and legume *NFR5* genes make them good candidates for testing the hypothesis that population diversities of partners in nitrogen‐fixing rhizobium–legume symbiosis are matched.

We tested the hypothesis on symbiotic systems of three wild‐growing legume species (*Vicia sativa*, *Lathyrus pratensis* and *Trifolium hybridum*) with their rhizobial microsymbionts. Sampling of the experimental material was performed uniformly on the one large natural fallow (more than 25 years) field in order to avoid the influence of ecological factors. We collected 30 plant individuals for each of three species and, for each individual, collected (a) leaves, (b) nodules, and (c) soil samples. After the pooling, we obtained nine samples (3 species × 3 types of materials) each containing aggregated information of 30 samples (Figure S1). *Vicia* and *Lathyrus* species represent the same cross‐inoculation group nodulated with *Rhizobium leguminosarum* bv. *viciae* strains, while *Trifolium* belongs to a separate group nodulated with *R. leguminosarum* bv. *trifolii*. One of the important traits of the rhizobium–legume symbiosis is the annual cycle of rhizobia, consisting of nodule formation with consequent amplification of rhizobia inside of the nodule, followed by a release of the nodule rhizobia back into the soil after nodule degradation leading to an increase of the frequency of this rhizobial genotype in the soil (Spaink, Kondorosi, & Hooykaas, 1998). Therefore, we analyzed both soil and nodule populations of rhizobia, which affect each other.

Testing the hypothesis of matching population diversities required comparison of structural (topological) characteristics of plant and rhizobia populations. Traditionally, the topological similarity between two populations is estimated as the congruence of two respective labeled trees (Leigh, Lapointe, Lopez, & Bapteste, 2011). Here, we propose a novel method to compare topologies of two gene trees with unlabeled leaves. The method is based on the gCEED approach (Choi & Gomez, 2009) that translates each population to the Gaussian mixture model in a K‐dimensional space. This method can be classified as a kind of beta‐diversity metric, which, by analogy with taxonomic (Jost, Chao, & Chazdon, 2011) and phylogenetic methods (e.g., UniFrac, Lozupone, Lladser, Knights, Stombaugh, & Knight, 2011), could be denoted as “topological beta‐diversity.” We apply it to show that the tree structures are concordant between the two symbiont species.

## 2 MATERIALS AND METHODS

### 2.1 Sampling

Three wild‐growing legume species (30 samples per species) together with rhizosphere soil—the common vetch *V. sativa*, the meadow vetchling *L. pratensis*, and the alsike clover *T. hybridum*—were uniformly collected from the large natural fallow (more than 25 years) field near the town Vyritsa (Gatchinskii region of Leningradskaya oblast, Russia, 59°24′7.74′′N; 30°15′28.74′′E). All sampled plants had formed nitrogen‐fixing symbiotic root nodules that were selected and thoroughly washed. Soil samples were collected from close proximity to the plant roots (1–5 cm). Despite the fact that a host plant has an influence on the rhizobial population within these soil samples, these samples are referred to as soil samples. Three nodules from each plant sample were picked from the main or the closest to the main lateral roots. For each legume species, we prepared three pools for DNA extraction: the plant pool (30 leaf pieces, 0.1 g each), the nodule pool (90 nodules, 3 nodules per plant individual), and the soil pool (30 soil samples, 0.2 g each).

### 2.2 *nod*A amplification and sequencing

DNA was isolated from soil and nodule pools by bead beating homogenization (Precellys 24) and purification (PowerSoil DNA Isolation Kit; MoBio). Two pairs of nested degenerate oligonucleotide primers were designed for *nod*A gene of *R. leguminosarum* bv. *viciae* and bv. *trifolii*. The first round of nested PCR with external primers—forward (5′‐DGGHYTGTAYGGAGTGC‐3′) and reverse (5′‐AGYTCSSACCCRTTT‐3′)—produces a 324 bp amplicon product; the second round with inner primers—forward (5′‐YTDGGMATCGCHCACT‐3′) and reverse (5′‐RDACGAGBACRTCTTCRGT‐3′)—produces a 217 bp amplicon product. The reaction conditions in the first and the second round of the PCR consisted of the initial denaturation step at 94°C for 3 min followed by 35 cycles with denaturation at 94°C for 30 s, primer annealing at 50°C for 30 s, and extension at 72°C for 1 min. The bar‐coded PCR products from six *nod*A libraries (soil and nodule libraries from three plants species) were sequenced with a Roche 454 GS Junior (following the manufacturer's protocols) generating an average of 3,000–4,000 reads per library. All obtained sequences were subjected to filtration by quality (quality score higher than 25), length (longer than 170 bp), and separating into libraries according to barcodes in QIIME. We introduced the term “pool” to designate a set of *nod*A gene sequences from nodule or soil rhizobia population. We performed the rarefaction analysis of the *π* nucleotide diversity within rhizobial pools to demonstrate whether the number of resultant sequences was sufficient to estimate the diversity (Figure S2).

The sequencing data were deposited at the NCBI short read archive under the bioproject number PRJNA297503. The multiple alignments of the remaining sequences within each pool were performed with ClustalW algorithm as implemented in MEGA (Tamura, Stecher, Peterson, Filipski, & Kumar, 2013). Sequences with frameshift errors were removed. The resultant multiple alignment for each pool did not contain gaps.

### 2.3 *NFR5* amplification and sequencing

DNA from plant leaf pools was isolated by AxioPrep kit (Axigen) and was used as the template DNA for PCR amplifications. Approximately 0.9 kb DNA fragments encoding all three LysM domains of the plant receptor gene, *NFR5*, were amplified with the following pairs of primers: forward “NFR5‐for4” (5′AAGTCTTGGTTGTTACTTGCC‐3′) and reverse “NFR5‐Grev3” (5′‐CACCTGAAAGTAACTTATCYGCA‐3′) for *V. sativa*; forward “NFR5‐for4” and reverse “NFR5‐Grev3” (5′‐TGCAGTCTCAGCTAATGAAGTAC‐3′) for *L. pratensis*; forward “NFR5‐for4” and reverse “NFR5‐Grev6” (5′‐CATACATTGTTGGCTTGCTTAC‐3′) for *T. hybridum*. The standard PCR protocol was used: initial denaturation at 95°C for 3 min, 30 cycles with denaturation at 94°C for 30 s, primer annealing at 48°C for 30 s, extension at 72°C for 1 min, and final extension for 4 min. PCR fragments were extracted from agarose gel (Onishchuk, Chizhevskaya, Kurchak, Andronov, & Simarov, 2015) and cloned into the plasmid pTZ57R/T (Thermo Scientific). For each plant species, 100 randomly selected cloned fragments of *NFR5* genes were sequenced by Sanger method in an automated ABI 3500xL sequencer (Applied Biosystems) using standard M13 (−20) and (−26) primers. Sequences were deposited in the GenBank database under the Pool accession number 1041522217. The multiple alignment of 100 sequences was performed with ClustalW as implemented in MEGA6 (Tamura et al., 2013).

### 2.4 Gene trees

The total *nod*A gene sequences from nodule and soil pools aligned with ClustalW were clustered into operational taxonomic units (OTUs) at 95% nucleotide sequence identity threshold using the UCLUST algorithm implemented in QIIME 1.9. A neighbor‐joining (NJ) dendrogram based on the numbers of differences between representatives of each OTU was constructed in MEGA6 and rooted using the outgroup *nod*A gene sequence of *Sinorhizobium meliloti* (GenBank ID AZNW01000092.1).

### 2.5 Diversity analysis

To compare the levels of nucleotide diversity *π* between plant and nodule rhizobia pools, we constructed the distributions of *π* statistics for each plant population subsampling with replacement of 70 sequences in 2,000 trials from each plant *NFR5* pool that initially contained 100 sequences. Subsequently, we randomly formed pairs of *π* diversity values from the obtained distributions for the plant pools and the respective nodule pools. The total set of 6,000 pairs (2,000 per plant sp.) was taken as a paired sample data. The relationship between host plants (*NFR5* gene) and nodule rhizobial (*nod*A gene) population diversity was assessed as the value of Spearman's rank correlation coefficient for the paired sample data. The values higher than 0.8 were taken to imply monotonic relationship in *π* between plant and nodule rhizobial pools.

All calculations were implemented in MATLAB. The link to the GitHub repository containing MATLAB scripts for the diversity analysis is provided in the Supporting information.

### 2.6 Statistical methods for detecting selection

The d*N*/d*S* ratio is a widely used measure to quantify the selection pressure acting on a set of homologous protein‐coding gene regions, where d*N* and d*S* are two measures of divergence between species, with d*N* corresponding to the number of nonsynonymous substitutions per nonsynonymous site and d*S* to the number of synonymous substitutions per synonymous site. The p*N* and p*S* statistics are analogous to the d*N* and d*S* statistics but are used for levels of polymorphism within a population rather than divergence (Kryazhimskiy & Plotkin, 2008). As we analyzed the gene regions that are smaller than the probable length of linkage disequilibrium blocks, we assumed that p*N* and p*S* value within this gene region can be nonindependent. Under the assumption of the nonindependence of p*N* and p*S*, the p*S* statistic does not reflect neutral mutations, and the p*N*/p*S* ratio became inconsistent. Thus, we analyzed p*N*, p*S*, and p*N*/p*S* separately, mostly focusing on p*N* as natural selection acts on nonsynonymous changes. Comparing the nodule and soil pools, we assumed that the increase of p*N* in one of them indicates the potential presence of stronger positive selection in it or the presence of stronger negative selection in the other pool. We hypothesized that the p*N* value in a soil rhizobial pool is greater than or equal to the p*N* value in the respective nodule pool. We tested this hypothesis for each legume species separately, performing Welch's *t* test considering 0.01 level of significance as described above.

Tajima's *D* statistic represents the difference between the observed and the theoretically expected nucleotide diversity (Tajima, 1989). If the mutations are neutral and the population adheres to the Wright–Fisher assumptions (Hartl & Clark, 2007), Tajima's *D* equals zero. Values significantly higher than zero indicate the deficit of rare alleles (e.g., due to a recent decrease in population size or balancing selection), and values significantly lower than zero indicate the excess of rare alleles (e.g., due to a recent population size expansion or purifying selection). In order to identify the selection type underlying the transformation of a soil pool to the respective nodule pool, we compared the values of Tajima's *D* between the pools. We considered the hypothesis that the Tajima's D in the soil pool is higher or equal to that in nodule pool. We tested this hypothesis using Welch's *t* test at 0.01 level of significance.

The calculations were performed using MATLAB PGEToolbox (Population Genetics Evolution Toolbox). The link to the GitHub repository containing MATLAB scripts for the analysis of selection is provided in the Supporting information.

### 2.7 Topological organization of diversity in plant and rhizobia pools

Let two populations of different sizes be represented by a set of aligned sequences. Let *p* be a population index, *p *∈ {1,2}. At the first step, the method identifies the unique haplotypes and their frequencies in each population, denoted as $\left\{\left(h_{i}^{p},f_{i}^{p}\right)\right\}$, $i=\bar{1,n_{p}}$ *,* where *n_p_* is the number of unique haplotypes in the population *p*. Let $D_{i,j}^{p}$ be the symmetric distance matrix between each pair of haplotypes in a population *p*, $i=\bar{1,n_{p}}$. At the second step, the hierarchical agglomerative clustering method merges haplotypes into clusters until the number of clusters equals to a predefined number *m*. We fix a population *p* and omit this index. The clustering algorithm starts with placing each haplotype in its own cluster that is described by two parameters: frequency *f_i_* and mean difference *σ_i_* (initialized to zero). Then, the following procedure is repeated until exactly *m* clusters occur. Two clusters, the *i*′‐th and *j*′‐th with the smallest pairwise distance ($i^{\prime },j^{\prime }:D_{i^{\prime },j^{\prime }}=\underset{i,j=\bar{1,n_{p}}}{\text{min}}D_{i,j}$), are merged into one new cluster. A distance from this cluster to a *k*‐th cluster is calculated as follows: $\frac{D\left(i^{\prime },k\right)f\left(i^{\prime }\right)+D\left(j^{\prime },k\right)f\left(j^{\prime }\right)}{f\left(i^{\prime }\right)+f\left(j^{\prime }\right)}$. The frequency of the new cluster is *f*(*i*) + *f*(*j*) and the mean difference of the new cluster is $D_{i^{\prime },j^{\prime }}$.

The described hierarchical clustering is applied to each population and yields the m clusters of haplotypes with the reduced distance matrix between them $D_{i,j}^{p}$, the frequencies $f_{i}^{p}$, and the mean differences within clusters $\sigma_{i}^{p}$, $i,j=\bar{1,m}$. In order to normalize the $D_{i,j}^{p}$ and $\sigma_{i}^{p}$ values between two populations, we divided these values by the median across $D_{i,j}^{p}$. If a cluster contains only one haplotype and its $\sigma_{i}^{p}$ is equal to zero, we set $\sigma_{i}^{p}=\underset{i=\bar{1,m};\;\sigma_{i}^{p}\neq 0}{\text{min}}\frac{\sigma_{i}^{p}}{f_{i}^{p}}$.

At the third step, the set of clusters for each population was translated into *K*‐dimensional Euclidean space (in the current project, we worked with 3D space) by Metric multidimensional scaling (Metric MDS) that transforms the distance matrix ${\left\{D_{i,j}^{p}\right\}}_{i,j=\bar{1,m}}$ into a set of coordinates ${\left\{x_{i}^{p}\right\}}_{i,=\bar{1,m}}$. Then, the Gaussian mixture model (GMM) is introduced for the population *p* as follows:

$$G_{p}\left(x\right)=\sum_{i=1}^{m}f_{i}N\left(x|x_{i}^{p},\sigma_{i}\right),$$

The adjustment of two GMMs is carried out by Procrustes superimposition: the minimization of the dissimilarity between mixtures (∆*G*) via translating, rotating, and mirror reflection. The lower the ∆*G* value is, the more similar two GMMs are and, consequently, the more similar the topologies of two population structures are. The exact formula to compute ∆*G* is presented in Choi and Gomez (2009; equation 12).

For each plant species, we performed two comparisons: “plant population versus nodule pool” and “plant population versus soil pool.” We used joint rhizobial nodule and soil populations before clustering and MDS, and separated them before the Procrustes analysis. This manipulation ensured that the same haplotypes in both comparisons were taken into account in the same way. We tested the null hypothesis that the ∆*G* value in the first comparison is greater than or equal to the ∆*G* value in the second comparison. In other words, the similarity between nodule rhizobia and plant pool topologies is not higher than the similarity between soil rhizobia and plant pool topologies. For each of the two comparisons, we obtained the set of ∆*G* values bootstrapping sequences in rhizobial pools. To test the hypothesis, we compared two obtained sets of ∆*G* values by one‐sided Mann–Whitney *U* test with 0.01 level of significance.

For visual comparison of plant and rhizobial populations, we constructed tanglegrams based on adjusted GMMs after Procrustes superimposition. A tanglegram is a diagram with a pair of two binary trees with matching leaves connected by edges. To construct it, we built two NJ gene trees for *m* plant *NFR5* clusters and *m* rhizobium *nod*A clusters based on the between‐cluster distance matrices and plotted two trees face to face. A pair of leaves from two trees was connected by an edge if a 3D point corresponding to one leaf was located within the five closest points to a point of another leaf and vice versa.

The link to the GitHub repository containing MATLAB scripts for topological beta‐diversity analysis is provided in the Supporting information.

## 3 RESULTS

### 3.1 *nod*A gene: OTUs and cluster analysis

**Figure 2:**
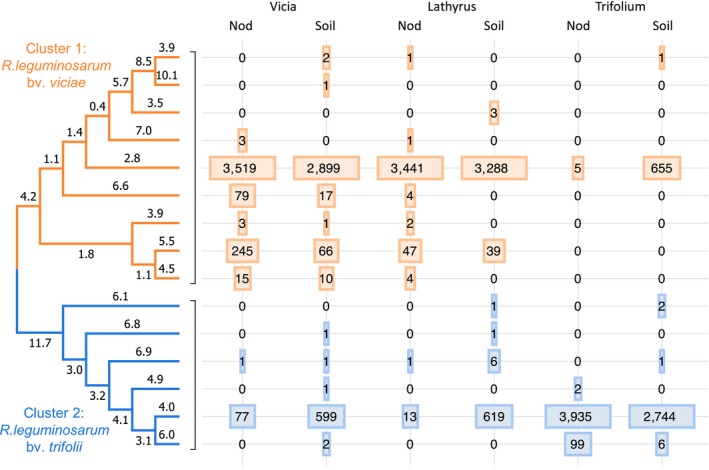
Clustering of *nod*A gene sequences after OTU‐picking analysis. Columns correspond to three plant species. Values in cells represent the numbers of OTU sequences in a pool, and widths of rectangles reflect the log of these values. The NJ tree of OTU‐representatives forms two clades corresponding to *Rhizobium leguminosarum* biovars from different cross‐inoculation groups: bv. *viciae* and bv. *trifolii*. Branch lengths indicate average nucleotide differences

*Nod*A gene libraries (for root nodules and for soil samples) were sequenced by NGS technology. Sequencing and filtration of *nod*A gene libraries produced a total of 22,463 sequences, for an average of 3,750 sequences per pool. Clustering by 95% sequence identity produced 15 OTUs, which frequencies differed between the pools. The NJ tree constructed for OTU representatives showed that the rhizobium population consisted of two clusters (orange and blue in Figure 2). Almost all sequences from the first cluster (9 OTUs) belonged to Vicia and Lathyrus pools, while sequences from the second cluster (6 OTUs) were mostly detected in Trifolium pools. The converse was also true: most of the Vicia‐Lathyrus pool belonged to the first cluster (90% average), while most of the Trifolium pool belonged to the second cluster. This distribution of OTUs was in agreement with the “cross‐inoculation groups” concept, which refers to the fact that separate groups of legume species can be successfully inoculated by only the specific groups of rhizobia. We attributed the first cluster to *R. leguminosarum* bv. *viciae* and the second to *R. leguminosarum* bv. *trifolii*. For further analysis, we kept in Vicia‐Lathyrus pool only sequences from the first cluster, and in Trifolium pools, only sequences from the second cluster. A small fraction of sequences in nodule pools (<5%) was from the improper cluster likely corresponding to a minor admixture of soil rhizobia adhered to the nodule surface.

### 3.2 Relationship between the bacteria and host plant diversities

**Figure 3:**
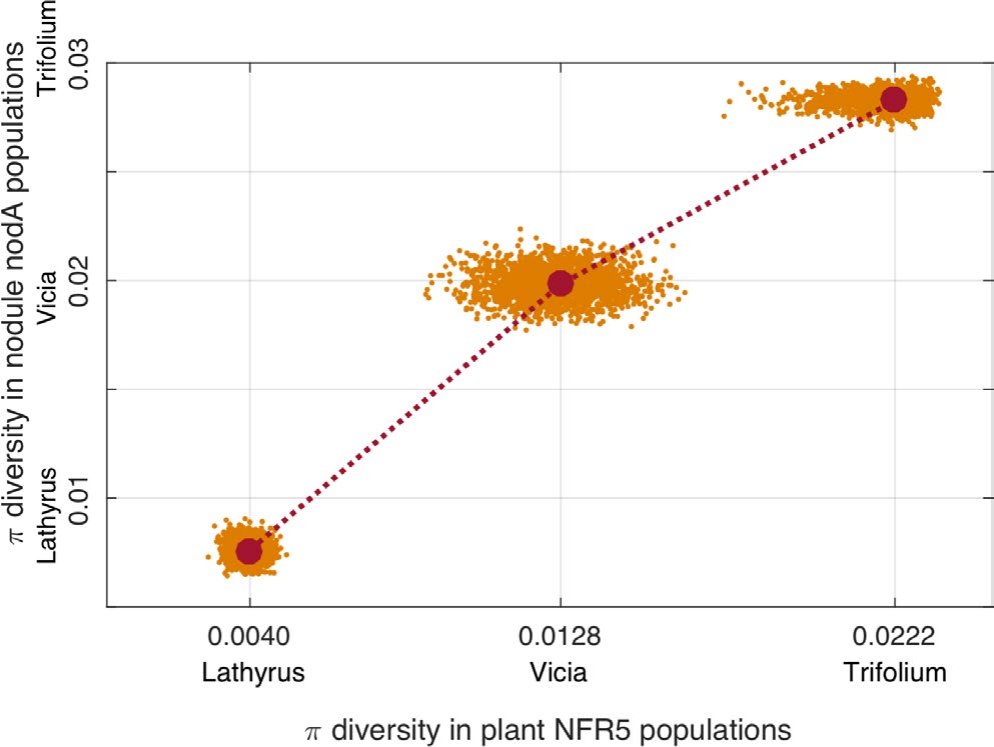
The monotonic relationship between the *π* diversity levels in nodule pools and plant pools. Dots represent the distribution of *π* obtained by bootstrapping

We calculated the diversity levels *π* in each plant population and found that the ranking of the three species was the same as the ranking based on the *π* nucleotide diversity values in corresponding nodule pools. By bootstrapping the plant and rhizobial nodule pools, we estimated the Spearman correlation between nucleotide diversities in these pools. The 0.89 value, which was higher than the predefined threshold, indicated that the monotonic relationship between diversities in pools of plant *NFR5* gene sequences and in bacterial *nod*A nodule pools is statistically significant (Figure 3).

### 3.3 Concordance of gene trees

A visual comparison of topologies of plant *NFR5* trees with those of corresponding rhizobial *nod*A trees for nodule and soil pools revealed that the topology of clades in the plant trees was more similar to the topology of clades in the nodule trees than in the soil trees (Figure S4, Appendix S1). Based on this observation, we proposed that the gene tree of the nodule rhizobia is more similar to that of the host plant than the gene tree of the soil rhizobia.

**Figure 4:**
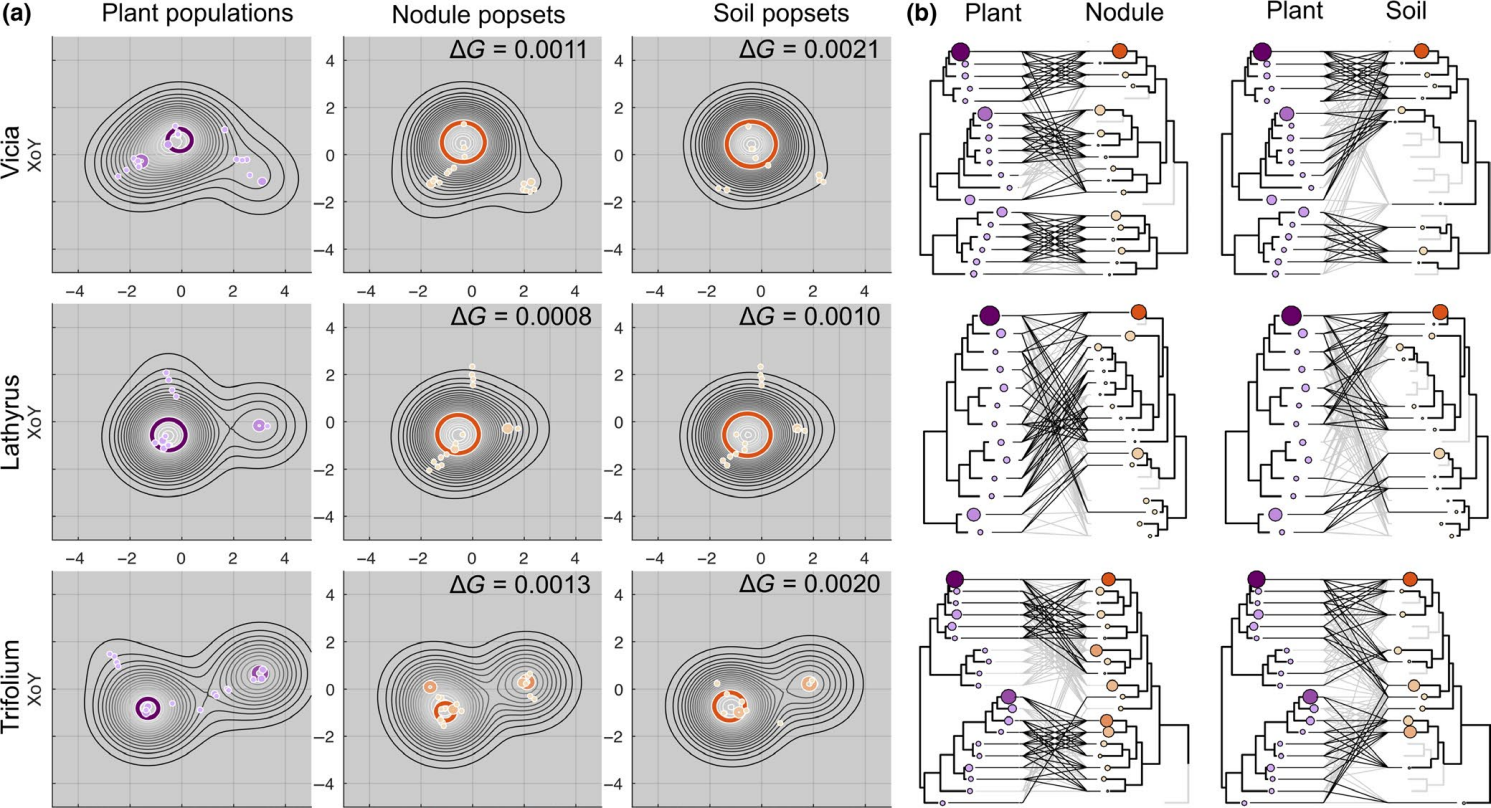
Comparison of plant pools with the bacteria pools from nodules and soil. (a) Projections of Gaussian mixture models for three plant *NFR5* pools and six rhizobial *nod*A pools after the Procrustes analysis on the XoY plane (see Figures S5–S7 for other projections). The values of ∆*G* correspond to the difference between the GMMs for plant and rhizobial (nodule or soil) pools; differences between ∆*G* values in each row are significant (*p* < .05). A visual comparison of projection confirms this trend. For example, in the “Vicia” row the rhizobium nodule pool has two peaks that refer to two peaks in the plant pool and are more distinct than in the rhizobium soil pool. (b) Tanglegrams for each plant species: between *NFR5* population and rhizobial *nod*A populations from nodule (left tanglegrams) and soil (right tanglegrams)

To formally test this, we developed a method for comparing structures (topologies) between two pools. We tested the null hypothesis that the topological similarity between a nodule rhizobia pool and a plant pool is not higher than the topological similarity between the soil rhizobia pool and the plant pool. This hypothesis was rejected at the 0.01 level of significance. We constructed tanglegrams that also illustrated a higher similarity of the topology of the nodule rhizobial *nod*A gene trees (Figure 4, left tanglegram) than the soil rhizobial *nod*A gene trees (Figure 4, right tanglegram) to that of plant NFR5 gene tree. The statistics ∆*G*, which reflects the topological difference between structures of plant and bacteria populations, can be referred as to “topological beta‐diversity.”

The obtained tanglegrams demonstrated that nodule pools contained clades of genotypes that were not detected in the corresponding soil pools (Figure 4). The presence of these clades can be a result of host plant selection of rare soil genotypes and is responsible for the increased topological similarity between *NFR5* gene pools with nodule *nod*A gene pools.

### 3.4 Analysis of selection

Analysis of population structures revealed the significant linkage between synonymous and nonsynonymous polymorphic sites, suggesting that the p*N* and p*S* statistics were not independent (see Appendix S2). Further comparison of p*N*/p*S* values between nodule and soil pools did not establish the significant difference (Figure S3). Despite the inconsistency of p*N*/p*S* in our case, we observed that the values of both statistics, p*N* and p*S*, were significantly increased (Welch's *t* test, *p*‐values < .01) in the nodule pools, indicating that both nonsynonymous and synonymous diversity were elevated there (Table 1). The values of Tajima's *D* were significantly lower than 0 in all of the rhizobial *nod*A pools, indicating the presence of negative selection (Table 1). However, within each plant, they were significantly higher in nodule pools than in the soil pools (*p*‐values < .01), suggestive of relaxation of negative selection or admixture of balancing selection in the former. *Trifolium* pools displayed the highest values of this statistic, consistently with the strongest admixture of balancing selection, while the *Lathyrus* pools were characterized by the lowest values.

**Table 1:** Values of p*N*, p*S*, p*N*/p*S*, and Tajima's *D* statistics for the pools of different origin. The difference in p*N*/p*S* values between nodule and soil pools for each plant was not significant

Pool	p*N*	p*S*	p*N*/p*S*	Tajima's *D*
Vicia				
Nodule	0.0170	0.0228	0.7450	−2.2644
Soil	0.0075**	0.0099	0.7600	−2.5219**
Lathyrus				
Nodule	0.0065	0.0086	0.7570	−2.5609
Soil	0.0051**	0.0072	0.7186	−2.5977*
Trifolium				
Nodule	0.0245	0.0335	0.7301	−2.0294
Soil	0.0167**	0.0232	0.7184	−2.2496**

** The significance (*p*‐value < .01) of the difference in values between nodule and soil population for each plant.

* Cases where .01 < *p*‐value < .05.

## 4 DISCUSSION

Symbiotic interactions represent a special case of ecological interactions when one of the partners provides an “environment” for another. In On the Origin of Species (Darwin, 1872), Charles Darwin proposed that “the life of each species depends in a more important manner on the presence of other already defined organic forms, than on climate.” This is particularly true for organisms in deeply integrated symbiotic systems.

Here, we traced the coordination in levels of population diversities between partners within the essential components of the rhizobium–legume signaling system: plant symbiotic receptor gene *NFR5* and *Rhizobium* symbiotic gene *nod*A involved in the synthesis of signaling molecules Nod factors (NFs), ensuring the first stage of partner recognition (Oldroyd, 2013). The matching was detected in two phenomena. The first is the monotonic relationship between the diversity of plant gene pools and the diversity of nodule rhizobial gene pools (Spearman correlation = 0.89). The second is the higher topological similarity between plant gene pool with nodule gene pool than with soil gene pool. The last phenomena were demonstrated using the developed method to compute “topological beta‐diversity”—the difference in topological structures of two population sets (plant and rhizobia) of gene sequences. The observed results allowed us to accept the hypothesis that population diversities of partners in nitrogen‐fixing rhizobium–legume symbiosis are matched.

We characterized the most pronounced characteristics of the mechanism underlying the matching population diversities. First, the analysis of the selection imposed by plants revealed significantly increased nonsynonymous diversity (p*N*) and Tajima's *D* values in the nodule gene pools (Table 1). This may be indicative of weaker negative selection in a nodule gene pool in a comparison with the respective soil gene pool, but is also consistent with a contribution of balancing selection or presence of stronger population structure in the former. In the previous study, it was shown that in symbiotic systems, besides the above‐mentioned types of selection, negative frequency‐dependent selection in favor of rare genotypes during the competition of rhizospheric bacteria for root nodulation (Amarger & Lobreau, 1982) may also play an important role (Andronov, Igolkina, Kimeklis, Vorobyov, & Provorov, 2015; Provorov & Vorobyov, 2000, 2006). Second, trees of nodule gene pools contained unique clades of genotypes, which were not detected in soil pools likely due to the low frequency of the genotypes in soil. These genotypes are probably responsible for increased topological similarity between plant and nodule rhizobia gene pools, so that a nodule population is selected by the host plant to supply some needs of the latter. In other words, our results demonstrated the transformation of the initial soil *nod*A pool by the template of the host plant receptor pool, and this conclusion is in line with the numerous works studying the interplay between diversities of host plant and rhizobia (Andronov et al., 2003; Bailly, Olivieri, Mita, Cleyt‐Marel, & Bena, 2006; Barrett, Zee, Bever, Miller, & Thrall, 2016; Bena, Lyet, Huguet, & Olivieri, 2005; Depret & Laguerre, 2008; Österman et al., 2011; Paffetti et al., 1998; Rangin, Brunel, Cleyt‐Marel, Perrineau, & Gilles, 2008; Vuong, Thrall, & Barrett, 2016). Finally, there are some suggestions on the molecular mechanism involved in this process. In our recent study, we modeled the 3D sandwich‐like structures of the *NFR5‐K1* heterodimeric receptor with its ligand Nod factor and observed the mutually polymorphic areas in the contact zone between *NFR5* and *K1* that were overlapped with known structural variation of Nod factor (in the fatty acid part) produced by *R. leguminosarum* bv. viciae (Igolkina, Porozov, Chizhevskaya, & Andronov, 2018). These results demonstrate the possible specificity of host plant receptors to variations in NF structure likely resulting in matching population diversities between *nodA* and *NFR5* gene pools.

The observed matching between nodule rhizobial *nod*A gene pools and plant *NFR5* receptor gene pools revealed the hierarchical organization of effective interaction: two symbionts should be genetically compatible at the single organism level and also at the population level. The process of forming this interaction could be explained metaphorically as an evolutionary molding: shaping the population structure of one symbiont using the population structure of another symbiont as a “matrix.” The important point in this shaping is the difference between evolutionary rates in plants and bacteria. The bacteria have a significantly higher evolutionary rate than plants; therefore, the diversity of the *nod*A gene in bacterial populations, like the flexible genetic material in the evolutionary molding, reflected the shape of more “rigid” diversity of the *NFR5* receptor gene in plant populations. We hypothesize that under the evolutionary molding effect, two symbiotic populations tend to relax the incoordination of genetic diversities between two parts of the symbiont–host signaling system, which is mostly achieved by a faster evolving partner, rhizobia in our case. We proposed that according to the effect described, the relationship in population diversity between rhizobia and host plant may be observed not only within the pair of *nod*A‐*NFR5* genes (which are related through the NF) but also within any pair of interplaying genes from plant and bacterial sides, and that genome‐wide scanning for “matching” genes can be an extension to the traditional methods of functional analysis of genes.

## CONFLICT OF INTEREST

None declared.

## Data Availability

NodA gene sequencing data were deposited at the NCBI short read archive under the bioproject number PRJNA297503. NFR5 gene sequences were deposited in the GenBank database under the Pool accession number 1041522217.
