# Supplementary material for: Matching population diversity of rhizobial nodA and legume NFR5 genes in plant–microbe symbiosis
Source: Ecol Evol. 2019 Aug 30;9(18):10377–86. doi: 10.1002/ece3.5556 (PMC6787799; doi:10.1002/ece3.5556)
Supplement: Supplementary file 1 [file ECE3-9-10377-s001.docx]

The link to the GitHub repository containing MATLAB scripts is https://github.com/iganna/popselection.git

**Supplementary figures**


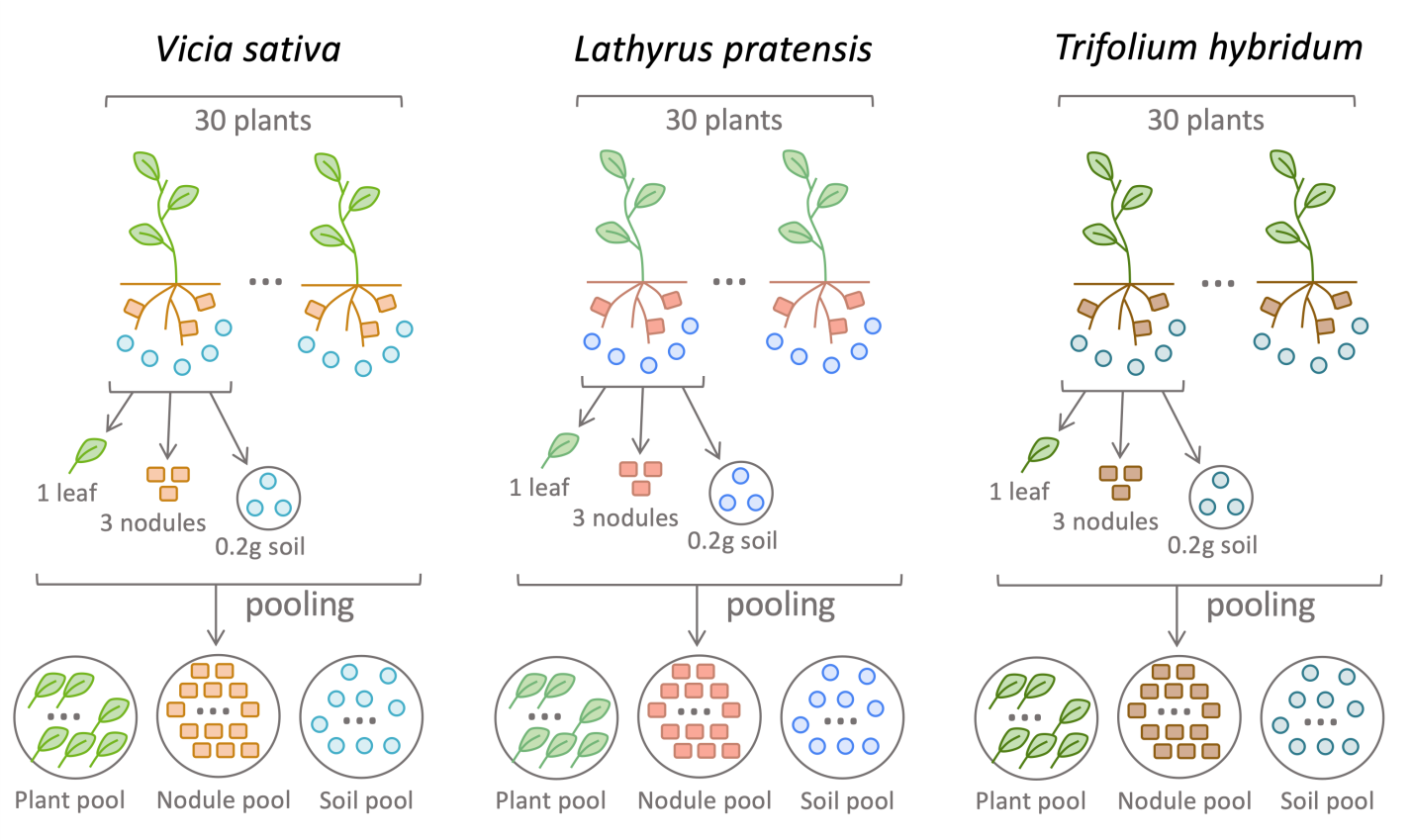


**Fig. S1.** The scheme of collecting the experimental material


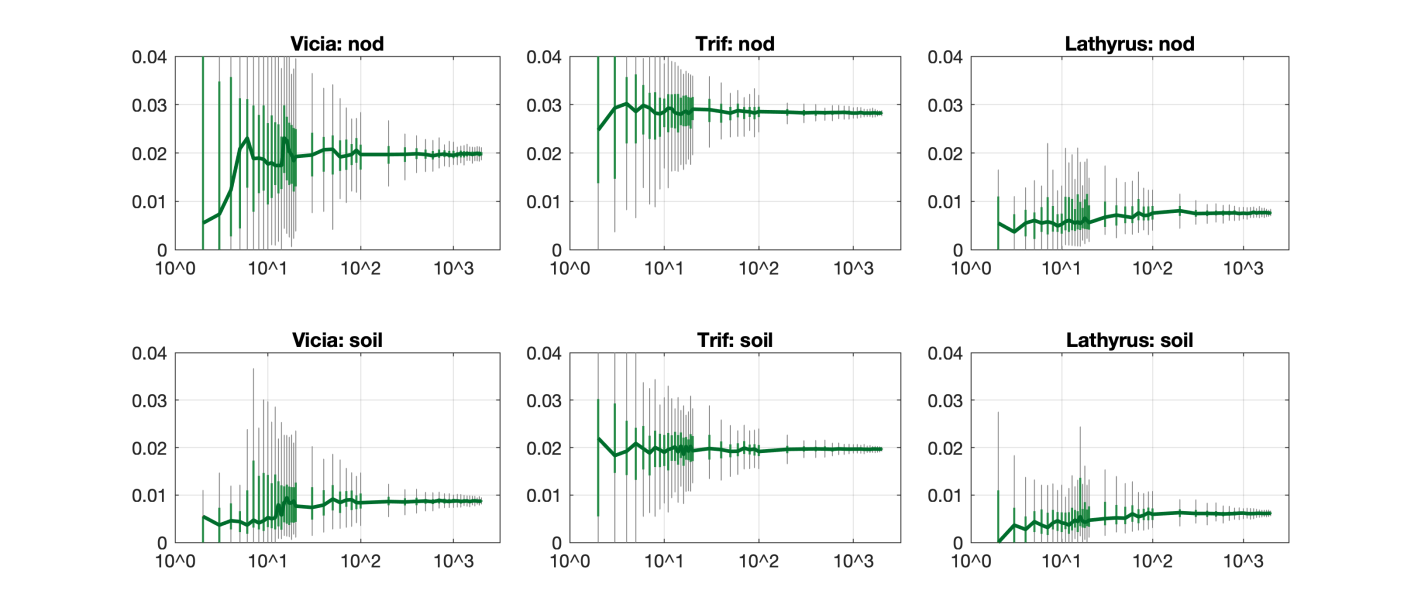


**Fig. S2.** Rarefaction analysis of nucleotide diversity in the analysed nodA gene pools.


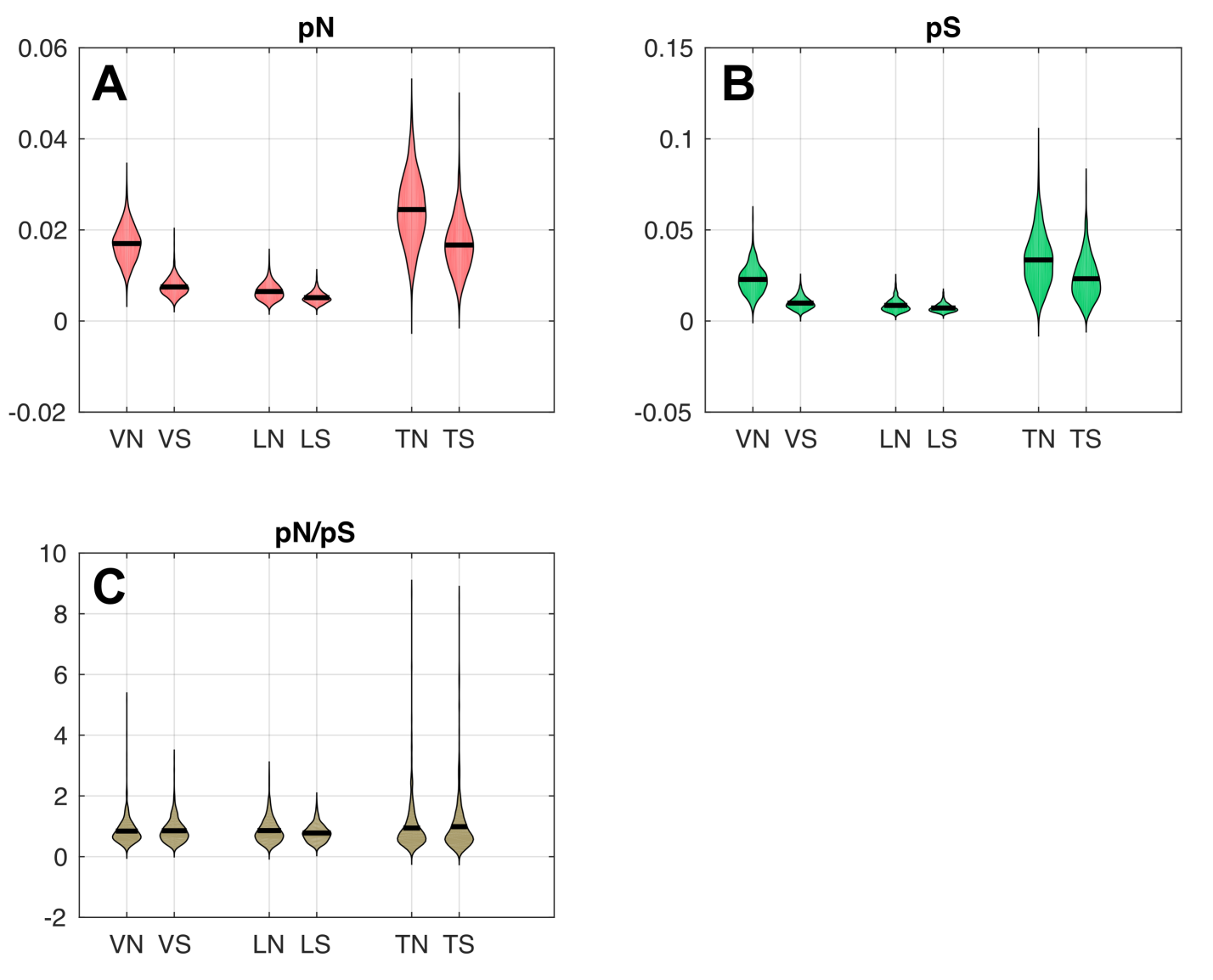


**Fig. S3.** Distributions of pN and pS statistics values. **(A,B)** Differences between in pN and pS values between nodule(N) and soil(S) nodA gene pools were significant (p-value < 0.01). **(C)** The difference in pN/pS values was not detected (p-value > 0.01). Letters “V”, “L”, “T” denote Vicia, Lathyrus, Trifolium nodA gene pools respectively. Letters “N” and “S” denote nodule and soil pools.


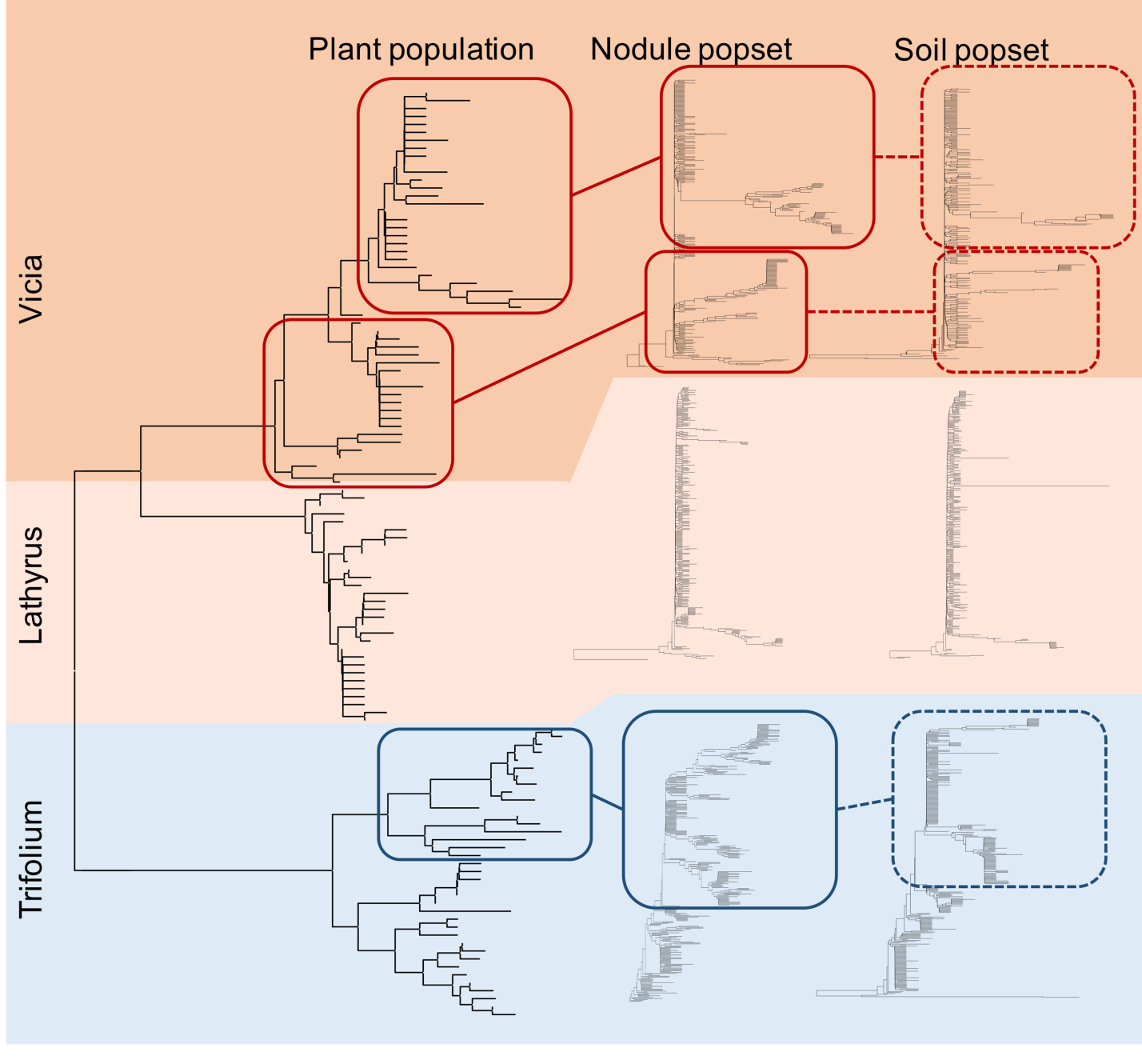


**Fig. S4.** Neighbour-joining (NJ) tree built for unique three plant haplotypes simultaneously contains three the major clades and is presented on the left. NJ for nodule and soil nodA gene pools was extracted from the NJ trees for joint (nodule+soil) pools with preservation tree topology, therefore, they are visually comparable. All the trees were rooted using outgroup sequences that were removed before visualisation.


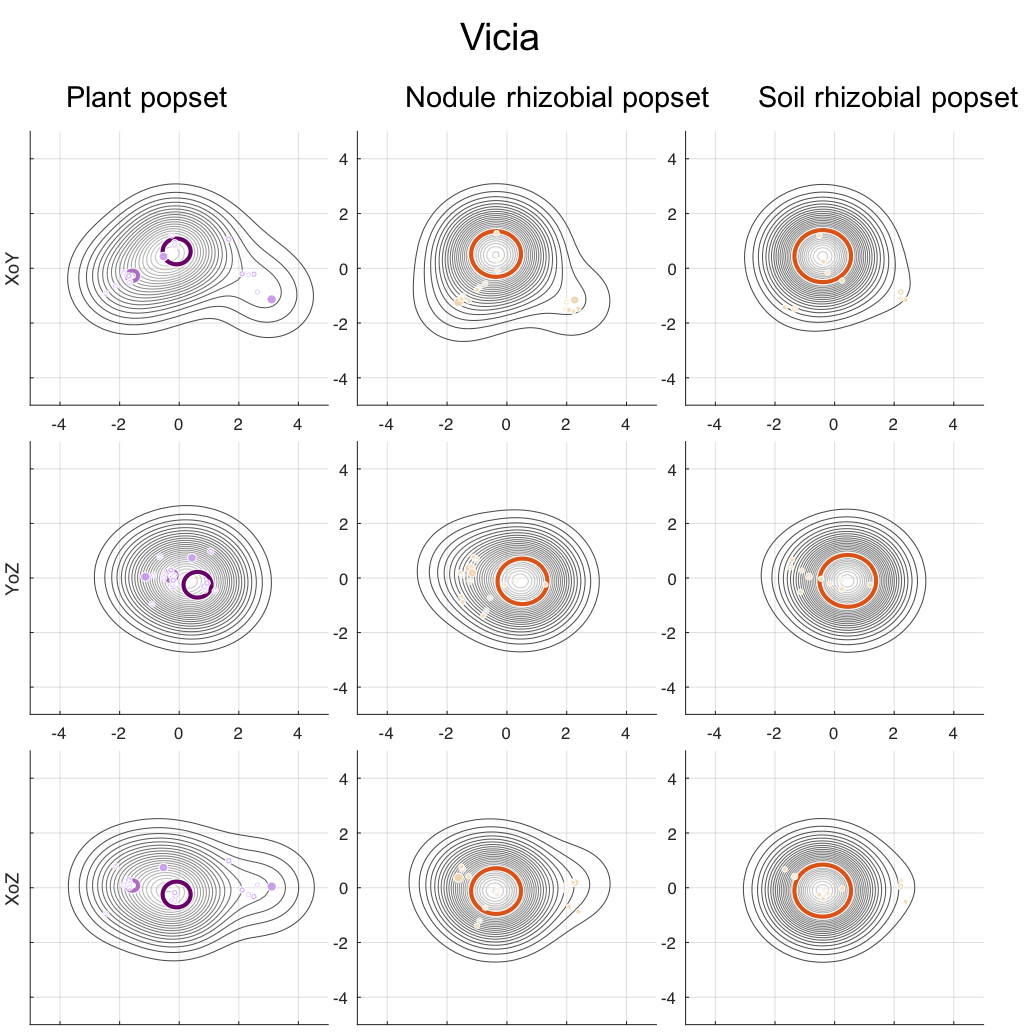


**Fig. S5.** Three projections of the Gaussian mixture models for Vicia host-plant NFR5 gene pool and Vicia rhizobial joint nodA gene pool (nodule and soil)


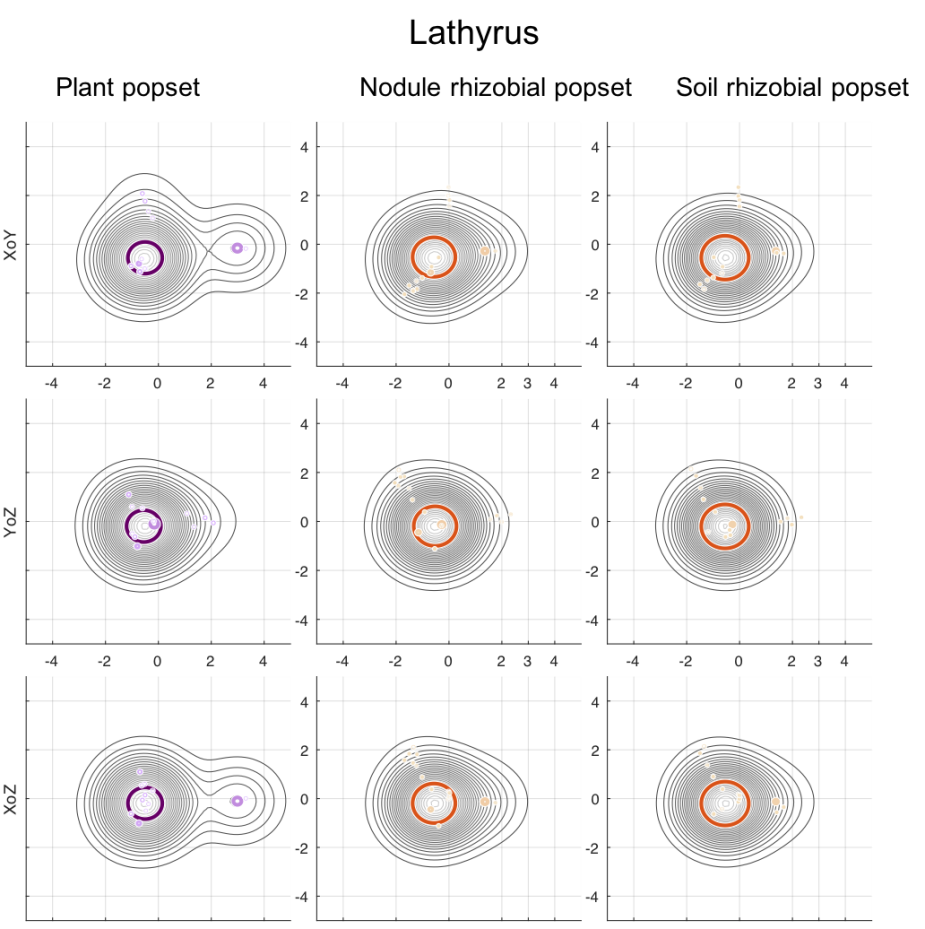


**Fig. S6.** Three projections of the Gaussian mixture models for Lathyrus host-plant NFR5 gene pool and Lathyrus rhizobial joint nodA gene pool (nodule and soil).


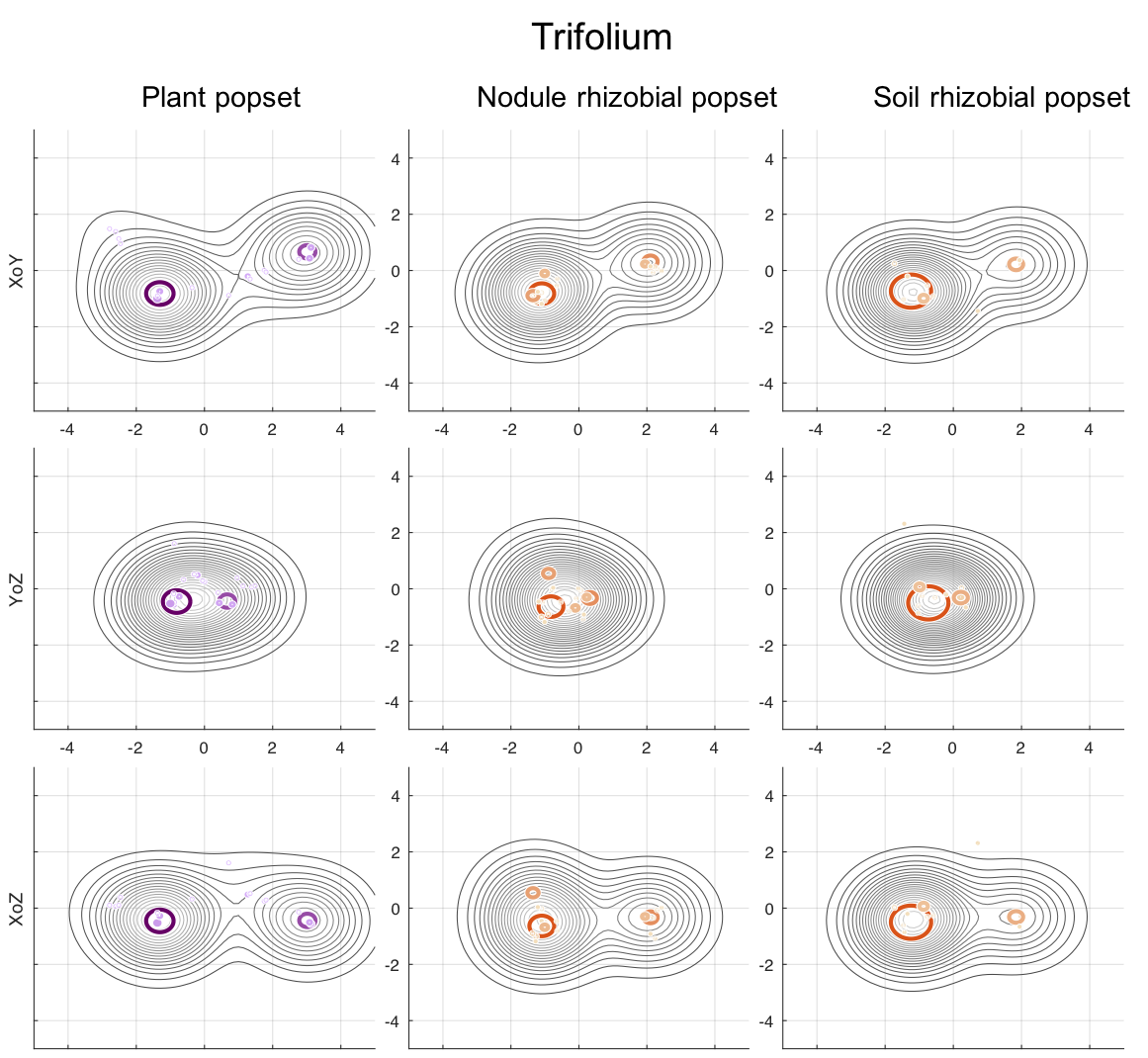


**Fig. S7.** Three projections of the Gaussian mixture models for Trifolium host-plant NFR5 gene pool and Trifolium rhizobial joint nodA gene pool (nodule and soil).

**Supplementary Texts**

**Appendix S1. Construction of gene trees for host-plant and rhizobial gene pools**

Detailed *nod*A gene trees for pools were obtained in three steps. For each plant species, we first combined nodule and soil *nod*A gene pools and identified unique haplotypes in the joint set of sequences. Next, we constructed a NJ dendrogram based on p-distances between all unique haplotypes and rooted it as above. Finally, we extracted two subtrees from the dendrogram according to the sequences from nodule and soil *nod*A gene pools. This algorithm yields structures of nodule and soil *nod*A gene pools that could be easily compared. Outgroups were removed from each tree before visualisation. The phylogenetic tree for *NFR5* gene sequences from all three plant species was also constructed using NJ algorithm based on p-distances.

**Appendix S2. Linked positions in nodA regions**

Within *nod*A region joint (nodule+soil) nodA gene pools for each legume species we tested all pairs of nucleotide positions to be significantly linked. We detected 24, 22 and 19 positions forming significantly linked pairs (chi-square test with pooling, BH adjusted p-value < 0.001) within Vicia, Lathyrus and Trifolium joint rhizobial pools, respectively. Overlapping between some linked pairs evinced the groups of linked positions that were confirmed after biclustering the symmetric matrix of mutual information between the positionsf.

We calculated pN and pS statistics at each codon containing detected positions and analysed the composition of identified groups. All groups of associated substitutions contained both non-synonymous and synonymous substitutions, to be more specific, each position with high pS in the corresponding codon placed in one group with at least one position with high pN in the corresponding codon. Moreover, there are several positions with high pN belonged to one group.

The groups of linked positions did not place on separate regions of *nod*A fragment, conversely, the nucleotide areas of the groups overlapped. When we compared sets of linked positions with the corresponding sets of informative positions we found them 98% overlapped.
